# *SYNE1* Exonic Variant rs9479297 Contributes to Concurrent Hepatocellular and Transitional Cell Carcinoma Double Primary Cancer

**DOI:** 10.3390/biomedicines9121819

**Published:** 2021-12-02

**Authors:** Yu-De Chu, Kwong-Ming Kee, Wey-Ran Lin, Ming-Wei Lai, Sheng-Nan Lu, Wen-Hung Chung, See-Tong Pang, Chau-Ting Yeh

**Affiliations:** 1 Liver Research Center, Chang Gung Memorial Hospital, Taoyuan 333, Taiwan; yudechu19871003@gmail.com (Y.-D.C.); victor.wr.lin@gmail.com (W.-R.L.); mingweilai@gmail.com (M.-W.L.); 2 Division of Hepatogastroenterology, Department of Internal Medicine, Kaohsiung Chang Gung Memorial Hospital, Kaohsiung 833, Taiwan; kee.kkm@gmail.com (K.-M.K.); juten@ms17.hinet.net (S.-N.L.); 3 Department of Hepatology and Gastroenterology, Linkou Chang Gung Memorial Hospital, Taoyuan 333, Taiwan; 4 Division of Pediatric Gastroenterology, Department of Pediatrics, Linkou Chang Gung Memorial Hospital, Taoyuan 333, Taiwan; 5 Whole-Genome Research Core Laboratory of Human Diseases, Chang Gung Memorial Hospital, Keelung 204, Taiwan; wenhungchung@yahoo.com; 6 Division of Urology, Department of Surgery, Linkou Chang Gung Memorial Hospital, Taoyuan 333, Taiwan; jacobpang@cgmh.org.tw; 7 Molecular Medicine Research Center, Chang Gung University, Taoyuan 333, Taiwan

**Keywords:** hepatocellular carcinoma, transitional cell carcinoma, single nucleotide polymorphism, spectrin repeat containing nuclear envelop protein 1, targeted exome sequencing

## Abstract

Unexpected high risk of synchronous/metachronous hepatocellular carcinoma (HCC) and transitional cell carcinoma (TCC) co-occurrence has been discovered previously. Here, we searched for genetic variation contributing to the co-occurrence of this double primary cancer (DPC). Using targeted exome sequencing, a panel of variants associated with concurrent DPC was identified. However, only a nonsynonymous variant within the *Spectrin Repeat Containing Nuclear Envelope Protein 1* (*SYNE1*) gene was associated with DPC occurrence (*p* = 0.002), compared with that in the healthy population. Further independent cohort verification analysis revealed that the *SYNE1*-rs9479297-TT genotype (versus TC + CC genotypes) was enriched in patients with DPC, compared with that in those with TCC alone (*p* = 0.039), those with HCC alone (*p* = 0.006), those with non-HCC/non-TCC (*p* < 0.001), and healthy population (*p* < 0.001). *SYNE1* mRNA expression reduced in both patients with HCC and TCC, and its lower expression in HCC was associated with shorter recurrence-free (*p* = 0.0314) and metastasis-free (*p* = 0.0479) survival. *SYNE1*-rs9479297 genotypes were correlated with tissue SYNE1 levels and clinical outcomes in HCC patients. Finally, *SYNE1* silencing enhanced the cell proliferation and migration of HCC/TCC cells. In conclusion, *SYNE1*-rs9479297 genotypes were associated with HCC/TCC DPC co-occurrence and correlated with *SYNE1* expression, which in turn contributed to HCC/TCC cell proliferation and migration, thereby affecting clinical outcomes.

## 1. Introduction

Hepatocellular carcinoma (HCC), a solid malignant tumor in the liver, ranks the sixth most common cancer type and the third leading cause of malignancies worldwide, with an estimated 800,000 newly diagnosed cases and 780,000 deaths occurring annually [1]. The ranking is even higher in Eastern and Southern Asia, including in Taiwan [1]. There are several known factors associated with HCC initiation and progression, including virus-related and non-viral causes, such as chronic hepatitis B or C infection, alcohol abuse, obesity, and metabolic diseases. In the past decades, the genomic alterations and instabilities have also emerged as novel underlying molecular mechanisms, which are either partially responsible for the consequences of these causes, or which serve as independent growth-promoting factors for HCC [2,3].

There are several well-documented curative strategies to treat HCC, including surgical resection and local ablation for patients with early-stage HCC, trans-arterial chemoembolization for patients with intermediate stage, and target therapy and immunotherapy for patients with advanced-stage [4]. Owing to the endeavor of early detection, advances in antiviral therapies for chronic hepatitis B and C, understanding of the oncogenic viral mutants, and success of novel anticancer strategies, survival in patients with HCC has greatly improved in recent years [5,6,7,8]. However, more than half of patients with newly diagnosed HCC are still in unresectable stages and have unsatisfactory therapeutic modalities. Furthermore, a number of them could develop extra-hepatic primary malignancy (EHPM), synchronously or metachronously, and liver cancer [9,10,11,12]. Recently, a large-scale pilot study in Taiwan, including 14,555 patients with HCC, revealed an EHPM rate of 3.91% (570 cases). Of the second primary cancer sites, the top three most frequent organs were the colon, kidney, and urinary bladder [9]. Intriguingly, although the incidence of colorectal cancer ranks first in Taiwan (as a single primary cancer), the ranks of kidney and urinary bladder cancers are far beyond the top 10 (14th to 17th depending on the year of ranking). An independent study consistently showed the same ranking order of the second primary cancer as EHPM in Taiwan [13]. Taken together, there is a disproportionally increased incidence of double primary cancer (DPC) comprising HCC and urinary tract cancer in Taiwan.

Globally, the annual incidence of newly diagnosed cases of bladder and kidney cancers was respectively 549,000 and 403,000, whereas the number of patients who died from these two malignancies was 200,000 and 175,000, respectively [1]. The majority of solid malignancies diagnosed in the kidney and bladder (or urinary tract) was transitional cell carcinoma (TCC), which is not a common cancer type in the world, including Eastern Asia or Taiwan [1]. Notably, the etiological factors of TCC are largely different from those of HCC according to epidemiological studies, except the use of aristolochic acid, which has been banned since 2003 in Taiwan [13] With the frequent co-occurrence of HCC and TCC, we suspect that there could be a common genetic factor leading to oncogenesis [9,13]. In this study, we aimed to identify any genetic variant that was associated with the co-occurrence of HCC and TCC DPC using a targeted exome sequencing (TES) approach followed by a functional link.

## 2. Materials and Methods

### 2.1. Patients and Samples

Various patient cohorts were included in this study. In the first cohort, 15 and 101 patients with HCC/TCC DPC were included for TES and validation analysis, respectively, using serum-derived genomic DNA. In the second cohort, 216 healthy Taiwanese individuals were included as a control group for comparison. Sequence data were retrieved from the Chang Gung Human database. A total of 44 patients with TCC, 265 patients with HCC, and 153 non-TCC and non-HCC subjects were included in the third cohort, and their serum-derived genomic DNA was assayed to examine the correlation between *SYNE1*-rs9479297 genotypes and DPC occurrence. In addition, 156 paired tissue samples (for RT-qPCR) and 20 paired tissue samples (for IHC staining) obtained from patients with HCC were included to assess *SYNE1* mRNA and protein expression levels in non-cancerous and cancerous tissues. All the patient information de-linked paired tissues and patient-derived blood samples were retrospectively obtained from the Tissue Bank, Chang Gung Memorial Hospital, under the permission of the institutional review board, Chang Gung Memorial Hospital, Linkou, Taiwan (201900261B0C101, 101-5246B, and 201600774BO), with written informed consent.

Finally, online reference datasets were acquired for the analysis of *SYNE1* mRNA levels in tissues from patients with HCC or TCC. In GSE14520, data from 126 normal and 130 cancerous liver tissues were retrieved accordingly. Data from 168 normal and 228 cancerous liver tissues were obtained in GSE63898. In GSE133624, data from 29 normal and 36 cancerous TCC tissues were retrieved accordingly. Lastly, data from 10 normal and 10 cancerous TCC tissues were used in GSE47702. Detailed information on the cohorts employed in this study is summarized in Figure S1, and the available baseline characteristics are listed in Table 1.

### 2.2. Targeted Exome Sequencing (TES)

Fifteen patients with HCC/TCC DPC were included in this analysis. Eighty nanograms of genomic DNA were amplified using four pools of 15992 primer pairs, the Ion AmpliSeq Comprehensive Cancer Panel (Thermo Fisher Scientific, Waltham, MA, USA, 4477685), to target all coding exons of 409 cancer-related genes (Table S1). Amplicons were ligated with barcoded adaptors using the Ion AmpliSeq Library Kit (Thermo Fisher Scientific, 4480441). Barcoded libraries were subsequently conjugated with sequencing beads by emulsion polymerase chain reaction (PCR) and enriched using Ion Chef^TM^ (Thermo Fisher Scientific, 4484177) according to the Ion Torrent protocol. The quality and quantity of the amplified library were determined using a fragment analyzer (AATI) and Qubit (Invitrogen, Waltham, MA, USA). Sequencing was performed on an Ion Proton sequencer (Thermo Fisher Scientific, 4476610) using the Ion PI chip (Thermo Fisher Scientific, A26770) according to the manufacturer’s protocol. The raw reads generated by the sequencer were mapped to the GRch37 reference genome using the Ion Torrent Suite (Thermo Fisher Scientific, version 4.2). The coverage depth was calculated using the Torrent Coverage Analysis plug-in (Thermo Fisher Scientific, version 4.2). Single nucleotide variants (SNVs) and short insertion/deletions were identified using the Torrent Variant Caller plug-in (Thermo Fisher Scientific, version 4.4). The Variant Effect Predictor (VEP) (Thermo Fisher Scientific, version 4.2) was used to annotate every variant with the database from COSMIC: version 70; dbSNP 138 and 1000 Genomes: phase 1. Variant coverage lower than 50 or a variant frequency lower than 5% were filtered out accordingly.

### 2.3. SYNE1-rs9479297 Genotyping

To examine the genotype of *SYNE1*-rs9479297, the nested PCR was performed. Two primers, SYNE1-F1: 5′-TGTGAAACCATGTTCTGTGCA-3′ and SYNE1-R1: 5′-TTGTGTGTGTGAGTTTGCGT-3′, were used for the first run. Five microliters of the first-run reaction product were used for the second-run reaction. The primers, SYNE1-F2: 5′-ATGTTCAGCTCCAGCTCAGA-3′ and SYNE1-R2: 5′-TCAAATGAGTGCACAGGCCA-3′, were used to perform the second-round PCR. A 251-bp amplicon was generated, and the gel-purified DNA product was subjected to direct sequencing to determine the genotype of *SYNE1*-rs9479297.

### 2.4. SYNE1-rs9479297 Genotyping

The IHC staining was conducted as described previously [14]. The recombinant anti-SYNE1 antibody (Abcam, Cambridge, UK, ab192234) was used for SYNE1 staining.

### 2.5. Cell Culture

Mahlavu and u1 cells were cultured in Dulbecco’s Modified Eagle Medium (DMEM) (Gibco, Waltham, MA, USA, 21969035) and Roswell Park Memorial Institute (RPMI) 1640 Medium (Gibco, 21870084), respectively, in a standardized culture environment, supplied with 5% CO_2_ in a humidified 37 °C incubator. Mahlavu cells [Research Resource Identifier (RRID): CVCL_0405] were kindly provided by Dr. Kwang-Huei Lin at Chang Gung University, and u1 cells (also called T-24 or MGH-U1, RRID: CVCL_0554) were a gift from Dr. See-Tong Pang at Chang Gung Memorial Hospital, Taoyuan, Taiwan.

### 2.6. Lentivirus-Mediated Knockdown of SYNE1

To achieve stable knockdown, lentivirus-mediated transduction of shRNA against *SYNE1* was conducted as previously described [15]. Briefly, packaging virion was conducted using HEK293T cells (RRID: CVCL_0063). The 1.3 μg shRNA, 1.1 μg pCMV-ΔR8.91, and 0.1 μg pMD.G were transfected into HEK293T cells using Maestrofectin (Omics Bio, New Taipei, Taiwan, MF002) according to the manufacture provided instructions. The pCMV-ΔR8.91 and pMD.G were purchased from RNAi core of Academia Sinica, Taiwan (Service ID: C6-1-1). The medium was replaced with 1% bovine serum albumin (BSA)-containing medium after 24 h incubation. The medium was collected to centrifuge and pass through the 0.22 μm pore size filter followed another 48 h incubation. The filtered medium with the generated lentivirus was harvested and aliquoted for subsequent experiments. The multiplicity of infection (MOI) used was approximately 100, either for Mahlavu or u1 cell. The medium was replaced with a fresh one containing 8 μg/mL polybrene (Sigma, St. Louis, MO, USA, H9268) after 16 h of cell seeding. The virion was directly added into the polybrene-containing medium. After 48 h incubation, cells were selected using 2 μg/mL puromycin (Gibco, A1113803). Following two-generation stabilization, the cells stably expressing shRNA were used for subsequent analysis. The target sequences of shRNA used to silence *SYNE1* were 5′-GCGTAGTGATAAGACTGATTT-3′ (clone ID: TRCN0000147281) for #1 and 5′-GCAGTTTAACTCAGACTTGAA-3′ (clone ID: TRCN0000147425) for #2. The control used in this assay was shRNA against LacZ, 5′-GCCGTCGTATTACAACGTCGT-3′ (clone ID: TRCN0000231700). All shRNA clones were purchased from the RNAi core of Academia Sinica, Taiwan.

### 2.7. Cell Proliferation Assay

The cell proliferation rate was assessed as previously described [15]. For each replicate, 3 × 10^3^ cells were seeded in each well of a 96-well plate. After 24 h post seeding, the Alarmar Blue cell viability reagent (Invitrogen, DAL1100) was directly added into the culture medium 3 h before cells were harvested for quantification of the fluorescent metabolites. The quantification assay was conducted daily until day 4.

### 2.8. Cell Migration Assay

Wound healing and transwell assays were used to assess cell migratory ability. The wound healing experiment was performed by using a wound healing assay kit (Abcam, ab242285) according to the manufacturer’s instructions. The procedures for transwell assay (Corning, Corning, NY, USA, CLS3422) were conducted as previously described [14].

### 2.9. RNA Isolation and Quantitative RT-PCR

RNA extraction was performed as previously described [16]. The ToolScript MMLV RTase (TOOLS, TGERA04) was used for reverse transcription according to the procedures provided by the manufacturer. The Bio-Rad CFX96 real-time system was used to detect the genes of interest. The *ACTB* qPCR primers used for quantitative RT-PCR were as follows: forward, 5′-CACCAACTGGGACGACATGG-3′, and reverse, 5′-AGGATCTTCATGAGGTAGTC-3′. *SYNE1* mRNA was assessed using the primers SYNE1-F2 as described in the *SYNE1*-rs9479297 genotyping section and the SYNE1-R3: 5′-TGACCTGTCAAATGCTTCGGT-3′.

### 2.10. Statistical Analysis

Parametric data with normal distribution are presented as mean ± standard deviation and were compared using Student’s *t*-test. Nonparametric data or data with non-normal distribution are expressed as medians (range) and were compared by Mann–Whitney test. The significance levels of associations were evaluated by either one or two degree-of-freedom 𝑥^2^ test. Survival analysis was performed by Kaplan–Meier analysis. Patients were divided into subgroups based on high and low levels for prognostic analyses according to the median of variable(s) or based on different *SYNE1*-rs9479297 genotypes. For post-hoc analysis, the Kruskal–Wallis test was performed followed with the Dunnett’s multiple comparison test for the non-parametric data, and the one-way ANOVA was conducted and followed with the Tukey test for multiple comparison test of parametric data.

## 3. Results

### 3.1. Identifying a Panel of Potentially Pathogenic Exonic Variants in Patients with HCC/TCC DPC

**Figure 1:**
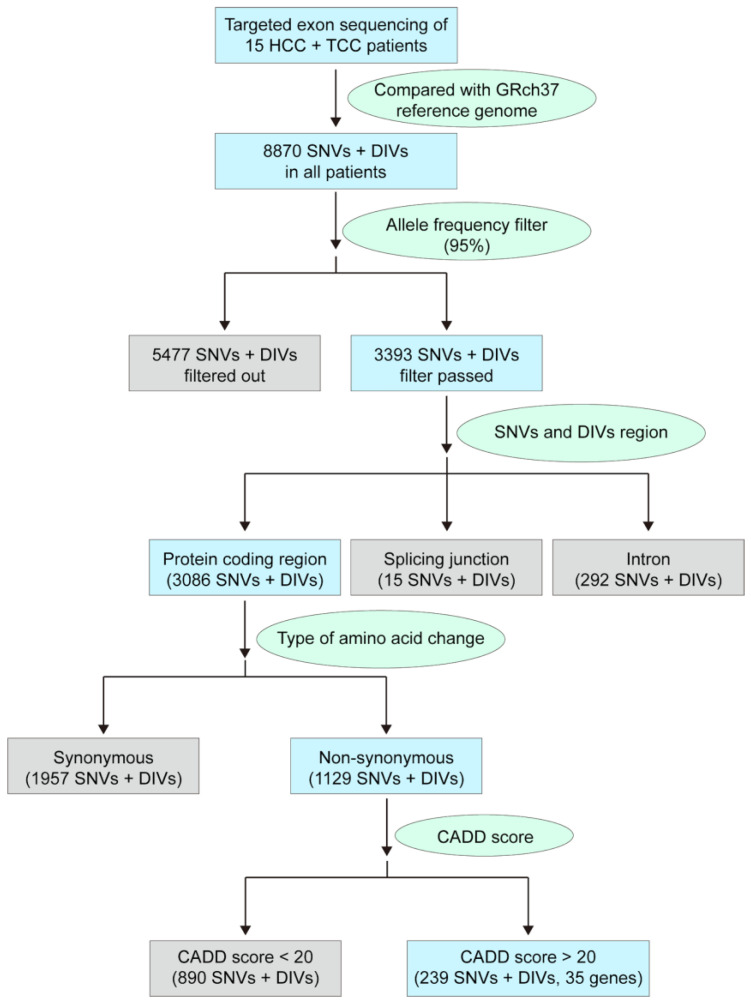
Flow chart of filtering criteria used in this study. HCC, hepatocellular carcinoma; TCC, transitional cell carcinoma; SNV, single nucleotide variation; DIV, deletion/insertion variation; CADD score, combined annotation-dependent depletion score.

A total of 15 patients, diagnosed with TCC, either before, synchronously, or metachronously to HCC, were included in this study. Overall, 4 (26.7%) patients had HCC developed before TCC, 6 (40%) patients had TCC developed before HCC, and 5 (33.3%) patients had both primary cancers diagnosed simultaneously. The time interval between the two cancers diagnosed ranging from 0 to 10 years. Their baseline characteristics are presented in Table 1. A total of 409 cancer-related genes were screened by using a TES approach (Table S1). All sequenced results were compared to those of the GRch37 reference genome; thus, 8870 variants, including SNVs and deletion or insertion variations (DIVs), were identified. As the filtering criteria depicted in Figure 1, the allele frequency was set as 95% to filter out the variants from somatic and/or heterozygotic mutations to understand whether any of the genetic markers from maternal derivatives could be an indicator of the occurrence of HCC/TCC DPC. Consequently, 3393 variants in these patients passed through the threshold. Next, in an attempt to identify any functional substitution or insertion/deletion in the protein-coding region that may affect its enzymatic activity and therefore be associated with DPC occurrence, the selection criterion was set to screen those that were not located in the protein-coding region, and 3086 variants remained accordingly. On further searching for the nonsynonymous amino acid change, 1129 variants that supposedly caused amino acid substitutions remained as candidates. Notably, the combined annotation-dependent depletion (CADD) score was further used to determine the deleterious effects of these variants [17]. Here, a CADD score of 20 was used as a cutoff, and 239 variants, including 35 genes with 44 SNP loci, remained as final candidates. Detailed information on these candidate variants is listed in Table 2, and the frequency and distribution of specific SNPs in individual patients are summarized in Figure S2A,B, respectively.

### 3.2. SYNE1-rs9479297 as a Potentially Pathogenic Allele for Occurrence of HCC/TCC DPC in Taiwan

These identified candidate genetic variants associated with HCC/TCC DPC were further tested to determine whether they were selected on account of racial differences in the genetic background between Taiwanese and the origin of the GRch37 reference. To address this issue, the genotype distribution of these variants in patients with HCC/TCC DPC was compared with that of a healthy population in Taiwan. As a result, most of the genotypic distributions of these variants identified from patients with DPC were found to be similar to those in the Taiwanese healthy population, indicating that they were attributed to racial differences (Table 3). However, among these SNPs, *SYNE1*-rs9479297 (*p* = 0.002), *SYNE1-rs76160752* (*p* < 0.001), *EPHA3-rs17801309* (*p* < 0.001), and *TRIP11-rs80200454* (*p* < 0.001) exhibited profound differences, compared with those of the healthy Taiwanese population (three-group comparison with variant homozygous, heterozygous, and reference homozygous), suggesting that the genotypic distributions of these four variants were significantly different from those of the GRch37 reference and Taiwanese healthy population. Subsequently, by further stratifying the cohorts into two groups (variant homozygous and others), only the genotypes of *SYNE1*-rs9479297 revealed marked significant differences, suggesting that the *SYNE1*-rs9479297 genotypes were associated with the occurrence of HCC/TCC DPC.

### 3.3. SYNE1-rs9479297-TT Genotype Associated with Occurrence of HCC/TCC DPC in Taiwan

To further verify whether *SYNE1*-rs9479297 could serve as a marker to predict the occurrence of HCC/TCC DPC, the genotypic distributions were compared among four independent cohorts of patients with HCC/TCC DPC (*n* = 101), HCC alone (*n* = 265), TCC alone (*n* = 44), and non-HCC/non-TCC (*n* = 148). In HCC/TCC DPC cohort enrolled for validation, 35 (34.7%) patients had HCC developed before TCC, 26 (25.7%) patients had TCC developed before HCC, and 40 (39.6%) patients had both primary cancers diagnosed simultaneously. The time interval between the two cancers diagnosed ranging from 0 to 13 years. As listed in Table 4, among the 101 patients with DPC, 12 had the *SYNE1*-rs9479297-TT genotype (11.8%, 95% coincidence interval 5.5–18.3%) and 89 had the non-TT genotype (72 and 17 for CC and CT genotypes, respectively), which was similar to the genotypic distribution of HCC/TCC DPC patients in the TES-analysis cohort (TT genotype, 33.3%, 95% coincidence interval 6.3–60.4%; Table 3). After genotyping for the other cohorts, it was revealed that none (0%) of the 44 patients in the TCC-only cohort (*p* = 0.039), 11 (4.2%, 95% coincidence interval 1.7–6.6%) of the 265 patients in the HCC only cohort (*p* = 0.006), and 2 (1.3%, 95% coincidence interval 0–3.1%) of the 153 patients in the non-TCC/non-HCC cohort (*p* < 0.001) were classified as the TT genotype, compared with the HCC/TCC DPC patients.

To determine whether there were any differences in the clinicopathological factors between patients with the *SYNE1*-rs9479297-TT genotype and non-TT genotype, the baseline characteristics of these patients were compared accordingly (for retrievable data from records, *SYNE1*-rs9479297-TT [*n* = 12] and non-TT [*n* = 74]). As listed in Table 5, there were no significant differences between the baseline data from patients with HCC/TCC DPC exhibiting rs9479297-TT and non-TT genotypes, except ages at diagnosis, which was significantly earlier in patients with the rs9479297-TT genotype (*p* = 0.0358 and 0.0067, for HCC and TCC, respectively). This finding suggests that the rs9479297-TT genotype is a genetic risk factor, rather than a factor associated with chronic liver or urinary tract diseases (i.e., chronic inflammation leading to cancers).

### 3.4. SYNE1 Expression is Downregulated in HCC and TCC and Associated with Clinical Outcomes in HCC Patients

**Figure 2:**
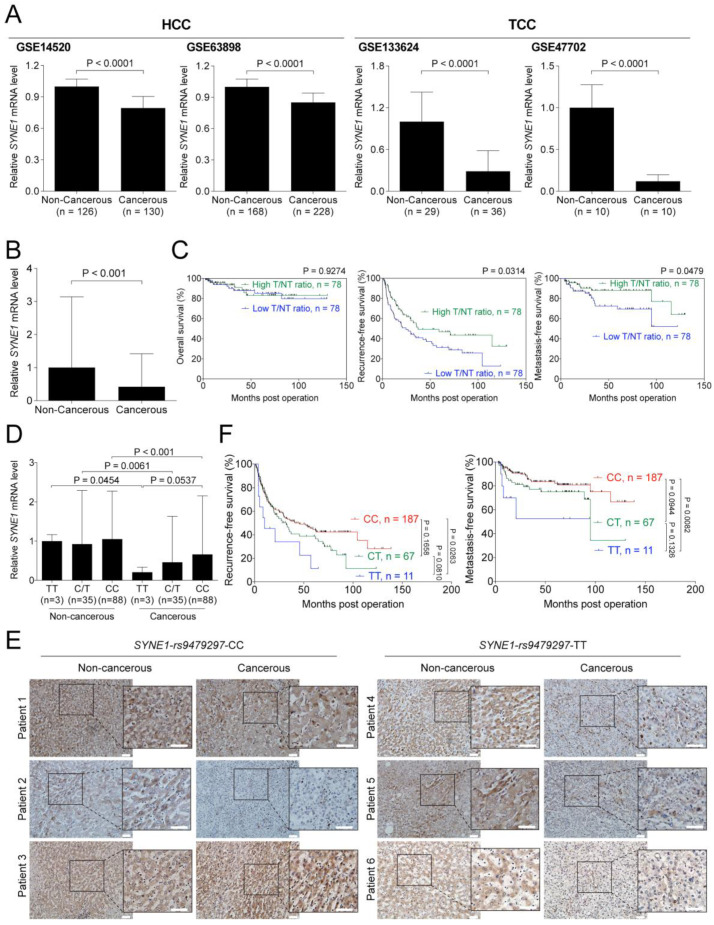
Rs9479297 genotypes associated with SYNE1 expression and patients’ clinical outcomes of patients. (**A**) The online available datasets of HCC, GSE14520 and GSE63898, and TCC, GSE133624 and GSE47702, were used to analyze the mRNA levels of *SYNE1* in HCC and TCC. (**B**) In total, 156 paired tumorous and nontumorous RNA derived from in-house patients with HCC were subjected into RT-qPCR analysis. (**C**) The median of the ratio of *SYNE1* mRNA in tumor divided by that in nontumor (T/NT ratio) assessed from (**B**) was utilized to separate patients into two groups. The Kaplan–Meier analysis of these two groups was then conducted. (**D**) In total, 126 patients enrolled in (**B**) were available for direct sequencing of rs9479297 genotypes and thus classified into three groups, according to the TT, CT and CC genotypes, for comparison of *SYNE1* mRNA expression. The *p*-values in (**A**,**B**,**D**) were obtained using the Kruskal–Wallis test and followed with the Dunnett’s multiple comparison test. (**E**) The IHC staining of tissues from HCC patients with rs9479297-TT or CC genotype. The white bar represents the scale bar, 50 μm. (**F**) In total, 265 HCC patients, as included in Table 4, were separated into three groups according to the rs9479297 genotypes, CC, CT and TT, and subjected into the Kaplan-Meier analysis.

To understand whether *SYNE1* levels were altered in HCC and TCC, in silico analysis was performed using four independent datasets, two for HCC (GSE14520 and 63898) and two for TCC (GSE133624 and GSE47702). As shown in Figure 2A, the expression levels of *SYNE1* mRNA were significantly decreased in cancerous tissues, compared with those in noncancerous tissues, either in HCC or TCC. Validation experiments were performed using RNA samples derived from the paired tumorous and nontumorous liver tissues of 156 in-house patients with HCC. Consistently, the *SYNE1* mRNA expression was markedly repressed in HCC (*p* < 0.001) (Figure 2B).

To examine the impact of altering *SYNE1* expression on clinical outcomes in patients with HCC, Kaplan-Meier survival curve analysis was performed. The patients were stratified into two groups, with high and low tumorous/nontumorous (T/N) ratios of *SYNE1* expression, using the median ratio as the cutoff. The overall survival (OS), local recurrence-free survival (RFS), and metastasis-free survival (MFS) in patients with HCC were estimated accordingly. As shown in Figure 2C, a higher *SYNE1* T/N ratio was correlated with better postoperative RFS and MFS, implying that lower *SYNE1* levels in cancerous tissue might enhance HCC recurrence and metastasis.

### 3.5. SYNE1-rs9479297 Genotypes Predict Postoperative Prognosis in Patients with HCC

To investigate the relationship between rs9479297 genotypes and *SYNE1* expression, the levels of *SYNE1* expression in HCC patients with TT, CT, and CC genotypes were compared. Among those enrolled in Figure 2B,C (156 patients with HCC), 126 had samples available for *SYNE1*-rs9479297 genotype examination. As shown in Figure 2D, there are 3, 35, and 88 patients identified as having *SYNE1*-rs9479297-TT, -CT, and -CC genotypes, respectively. Consistently, the *SYNE1* mRNA levels were reduced in the tumorous parts of patients with distinct genotypes (*p* = 0.0454, 0.0061, and < 0.001 for TT, CT, and CC genotypes, respectively). Interestingly, the lowest *SYNE1* level was observed in the cancerous parts of those with the TT genotype, although it was under a borderline significance (compared with those with the CC genotype in the cancerous parts, *p* = 0.0537).

To confirm this finding, IHC staining was performed to examine the protein levels of SYNE1 in tissues derived from patients with HCC. A total of 20 patients, 10 with the CC genotype and 10 with the TT genotype, were included in this analysis. Among those with the CC genotype, five showed decreased SYNE1 levels in the tumorous parts as representatively demonstrated in Patient 2, whereas two of the remaining five patients exhibited the same levels (as in Patient 1) and the remaining had higher SYNE1 levels (as in Patient 3) (Figure 2E, left panel). However, of the 10 patients with the TT genotype, 9 displayed lower SYNE1 levels in the tumorous part, and only one patient showed the same level as in the nontumorous parts (Figure 2E, right panel). The difference between these two groups (TT versus CC) was significant (*p* = 0.0246, tested by 𝑥^2^ distribution, density assessed using a computer program), suggesting that the rs9479297 genotypes were associated with SYNE1 expression levels, at least in patients with HCC.

Accordingly, the relationship between the genotypes and clinical outcomes in patients with HCC was addressed. A total of 265 patients with HCC in Table 4 were included in this analysis. As demonstrated in Figure 2F, patients with HCC harboring the TT and CC genotypes showed profound differences in RFS (*p* = 0.0263) and MFS (*p* = 0.0082), in which those with the TT genotype correlated with unfavorable survival. Similarly, in 87 HCC/TCC DPC patients with available prognostic data, patients with the CC genotype (*n* = 63) had a significantly better OS than those with the TT genotype (*n* = 12, *p* = 0.0288). Additionally, patients with the CC genotype had a better RFS than those with the TT genotype (*p* = 0.0217) (Figure S3A). On the other hand, when assessing the correlation between *SYNE1*-rs9479297 genotypes and prognosis according to TCC progression, no significant association was found in either patients with HCC/TCC DPC or those with TCC alone (Figure S3B,C).

### 3.6. SYNE1 Silencing Promotes Cell Proliferation and Migration in HCC and TCC Cells

**Figure 3:**
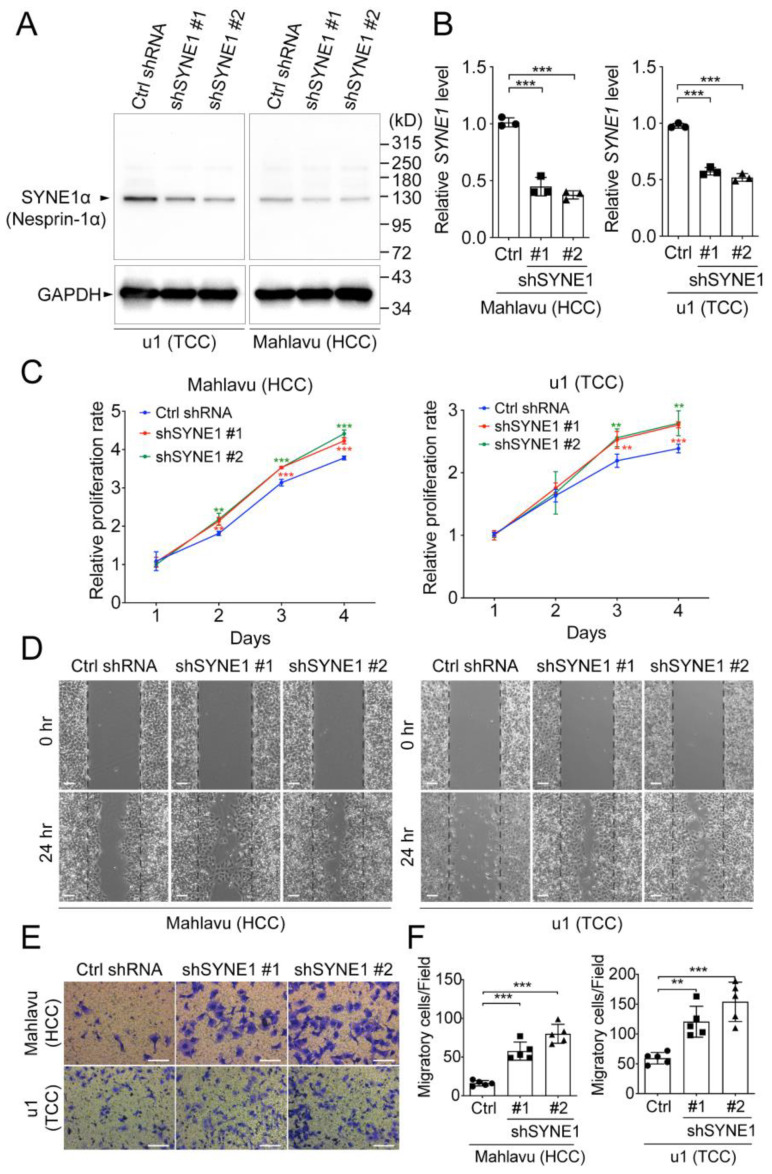
SYNE1 silencing enhanced HCC and TCC cells proliferation and migration. The HCC (Mahlavu) or TCC (u1) cells with or without depletion of SYNE1 were assayed to determine the SYNE1 levels by (**A**) Western blot analysis and (**B**) RT-qPCR analysis. *n* = 3 for each experiment. (**C**) The Alarmar Blue-based cell viability assay was performed to assess the cell renewal ability in cells with or without SYNE1 silencing. The (**D**) wound healing assay and (**E**) transwell-based assay were employed to examine the migratory capability of cells with or without SYNE1 knockdown. The white bar represents the scale bar, 250 μm. The quantification of migratory cells in transwell-based assay was shown in (**F**). For each experiment, the migratory cells in five independent capturing fields were counted accordingly. ** *p* < 0.01, *** *p* < 0.001. The *p*-values were acquired using the one-way ANOVA, followed with the Tukey test for multiple comparison test.

To examine the growth regulatory roles of SYNE1 in HCC and TCC cells, SYNE1 knockdown experiments using lentivirus-mediated downregulation were conducted. Two independent shRNA clones, #1 and #2, were obtained and their efficiencies to knockdown SYNE1, either in HCC (Mahlavu) or in TCC (u1) cell lines, were evaluated accordingly (Figure 3A,B). Consistently, it was found that the SYNE1-α (an isoform expressed from another alternative transcription start site with a molecular weight of approximately 125 kDa, also named as Nesprin-1α) was the predominant isoform in HCC cells (Figure 3A) [18]. To determine the cell proliferation rates upon SYNE1 silencing, cell growth curves were assessed and compared between SYNE1 knockdown cells and controls, in Mahlavu and u1 cell lines. As shown in Figure 3C, suppression of SYNE1 expression significantly enhanced cell proliferation in both HCC and TCC cell lines. Moreover, reduction in SYNE1 expression markedly promoted cell migration in Mahlavu and u1 cells using either wound healing assays (Figure 3D) or transwell-based experiments (Figure 3E,F).

## 4. Discussion

HCC is mainly caused by chronic hepatitis B and C infections in Asia. As such, the non-viral-related, genetic factors responsible for hepatocarcinogenesis are very difficult to identify. Here, we took advantage of the unproportionally high incidence of HCC/TCC DPC in Taiwan to explore this issue. In this study, the *SYNE1*-rs9479297-TT genotype was identified as a predictor of co-occurrence of HCC/TCC DPC (approximately 12% versus 2% in the healthy population). Furthermore, it was associated with unfavorable clinical outcomes in patients with HCC, at least in part, by altering SYNE1 expression levels, thereby affecting cell proliferation and migration. Thus, *SYNE1* gene might play a role in non-viral-related hepatocarcinogenesis. The rs9479297 genotypes have never been referred to as a predictor of disease occurrence or clinical prognosis in any other disease to date, albeit other genetic polymorphisms within *SYNE1* gene have been implicated in numerous cancer types, other than HCC or TCC [19,20,21,22,23,24,25].

The concurrent occurrence of HCC and EHPM has been widely considered a consequence of older age as mentioned in previous studies conducted in Taiwan [9,13]. Nevertheless, a sustained proportion of patients were diagnosed with DPC at a younger age (approximately 10% of total patients with HCC and EHPM, <60 years old) from 1986 to 2013 in Taiwan, for unknown reasons [9]. One proposed possibility for these patients with early development of HCC and EHPM might be due to pre-existing genomic mutations or variants. Our results supported this view, as patients with the *SYNE1*-rs9479297-TT genotype were diagnosed with HCC/TCC DPC at an earlier age, compared with those with non-TT-genotypes (Table 5).

The genetic variant of rs9479297, was located in the 75th exon of *SYNE1* gene, with a T-to-C substitution, resulting in a K4050R amino acid change. In Taiwan, the majority of people carry arginine at residue 4050, i.e., the CC genotype is the predominant one, whereas the TT genotype (the minor form) is associated with HCC/TCC DPC and poorer prognosis in HCC. At this time, it is not clear whether the R-to-K substitution leads to a functional change in the SYNE1 protein. However, it was interesting to find that different rs9479297 genotypes were correlated with distinct degrees of SYNE1 expression at both mRNA and protein levels (Figure 2D,E). A previous report demonstrated that a long segment (over 500,000 bp) within *SYNE1* was identified, manifesting a high acetylation status in chromatin and possibly playing a crucial role in modulation of the expression of *SYNE1* and/or other adjacent genes in leiomyoma [26]. As the identified DPC co-occurrence-associated SNP was located within the same long segment and the TT genotype correlated with both mRNA and protein levels of SYNE1, it could be speculated that the rs9479297 genotypes contribute to modulation of the transcription activity of *SYNE1*. As shown in Table 3, there is another SNP variant located in the *SYNE1* (rs76160752), associated with HCC/TCC DPC. Together, these variants may affect the chromatin acetylation status of this modulatory segment. In addition, multiple protein isoforms of SYNE1 have been reported to be generated from the same gene but with different transcription start sites and independent stop codons. Although the *SYNE1* transcripts have been reported by the Genotype-Tissue Expression (GTEx) project to be relatively lower in abundance in the liver tissue [27], this finding has not been completely reflected in the protein levels, as demonstrated by The Human Protein Atlas Project [28]. The predominant isoform in liver and HCC cells is SYNE1-α (Nesprin-1α) [18,29,30]. The rs9479297 containing long segment is just located in front of the transcription start sites of either SYNE1-α and -β isoforms, further supporting that this long segment might be crucial for transcriptional regulation of SYNE1 and/or its isoforms.

Pathologically, the effect of aberrant expression or mutations located within the C-terminus of *SYNE1* have been extensively investigated in the literature. They were found to be associated with the incidence of nerve and muscle diseases, such as cerebellar ataxia [31], albeit the detailed molecular mechanisms remain unclear. Several reports have indicated that in various cancers and tumors, including lung cancer, colorectal cancer, and serrated adenomas, *SYNE1* expression is epigenetically repressed through hypermethylation of the CpG island, residing in either the promoter or coding region [32,33,34]. Increasing evidences has demonstrated that reduced expression of SYNE1 leads to abnormalities in cell morphology, due to its functional role in establishing the linker of nucleoskeleton and cytoskeleton (LINC) complex by regulating its association with other proteins, such as SUN1/2, UNC84, and SPAG4 [35]. These processes control the shape and positioning of the nucleus. Impairment in the expression level or full functionality of the LINC complex leads to development or disease progression in numerous types of cancers [35]. Dysregulation, dysfunction, or mutation in SYNE1/Nesprin-1 plays an important role in oral cancer [36], glioblastoma [24], breast cancer [37], ovarian cancer [19] and HCC [38,39], possibly through the regulation of cell proliferation and apoptosis [40]. A report published by Shaglof et al. had demonstrated that one rarely studied isoform derived from alternatively spliced variants of SYNE1/Nesprin-1, named X6, was elevated in HCC-bearing rats, which might play a role in hepatocarcinogenesis and cancer progression [39]. Our present results are consistent with most of the published literature, showing that lower SYNE1 expression is correlated with more aggressive cancer phenotypes. This may be caused by reduced LINC complex function due to repressed SYNE1 expression. Recent studies have shown that alterations in the nuclear envelope and LINC complex function might directly contribute to tumorigenesis, as disrupted nuclear envelope structures render the cells more prone to dysregulated mitosis [24,35,36,38].

Most of the SYNE1 isoforms were reported to be predominantly located at the nuclear membrane to serve as scaffolds linking the nucleus and cytoplasmic cytoskeletons [18,29,30]. Under specific conditions [41], they can translocate to the nucleus or nucleolus. However, to date, there have been no reports regarding the pathological roles of the nucleus-localized SYNE1 isoforms. Notably, albeit the number of cases was relatively small, the images shown in Figure 2F, further demonstrate that in HCC patients with the rs9479294-TT genotype, the predominant IHC staining signals are present in the cytoplasm of the noncancerous section, which is consistent with the known function of SYNE1, serving as a member of the LINC complex. Yet, it was observed that in cancerous tissues, there were both cytoplasmic and nuclear staining of SYNE1, at least in two out of three HCC patients with the rs9479294-TT genotype (Patient 4 and 6). In combination with the findings from this study that HCC patients with the rs9479294-TT genotype had unfavorable prognoses, it could be postulated that the nucleus-localized SYNE1 can play a growth-promoting role in HCC (besides the disrupted LINC complex function), although further investigations are needed in this regard.

## 5. Conclusions

In conclusion, the genetic variant of *SYNE1*-rs9479297 was identified and was found to be associated with the occurrence of HCC/TCC DPC. The SNP was associated with the expression level of SYNE1, which in turn modulated HCC and TCC cell proliferation and migration, thereby affecting the clinical outcomes of patients with HCC alone or with HCC/TCC DPC.

## Figures and Tables

**Table 1:** Baseline characteristics of all retrospectively enrolled subjects in this study.

Cohort	Cohort-1	Cohort-1	Cohort-2	Cohort-3	Cohort-3	Cohort-3
Case number	*n* = 15	*n* = 101	*n* = 216	*n* = 44	*n* = 265	*n* = 153
Disease	DPC	DPC	Healthy	TCC	HCC	Non-HCC Non-TCC
Experiment or assay	TES	Rs9479297 genotype comparison	Rs9479297 genotype comparison	Rs9479297 genotype comparison	265 for Rs9479297 genotype comparison; 156 paired noncancerous and cancerous tissues for RT-qPCR; 20 paired noncancerous and cancerous tissues for IHC	Rs9479297 genotype comparison
*Baseline characteristics*						
Gender, male, *n* (%)	11 (73.3%)	65 (64.4%)	108 (50.0%)	34 (77.3%)	211 (79.6%)	91 (59.5%)
Age of sampling ± SD	59.3 ± 11.9	65.7 ± 9.3	70.6 ± 9.5	59.0 ± 8.9	54.8 ± 14.3	71.9 ± 6.0
HCC Ages (years) ± SD	59.3 ± 11.9	65.7 ± 9.3	NA	NA	54.8 ± 14.3	NA
TCC Ages (years) ± SD	59.5 ± 14.0	65.2 ± 9.6	NA	59.0 ± 8.9	NA	NA
Sequential order score ± SD ^a^	2.1 ± 0.8	1.9 ± 0.8	NA	NA	NA	NA
Body weight ± SD	65.0 ± 15.5	62.5 ± 11.7	NA	NA	NA	NA
Body height ± SD	164.7 ± 9.1	161.2 ± 7.5	NA	NA	NA	NA
Heavy smoker, *n* (%)	7 (46.7%)	41 (40.6%)	NA	NA	NA	NA
BCLC stage for HCC, n (%) Stage 0 Stage A Stage B Stage C Stage D	4 (26.7%) 7 (46.7%) 4 (26.7%) 0 (0%) 0 (0%)	15 (14.9%) 23 (22.8%) 20 (19.8%) 42 (41.5%) 1 (1.0%)	NA NA NA NA NA	NA NA NA NA NA	0 (0.0%) 8 (3.0%) 88 (33.2%) 144 (54.3%) 25 (9.4%)	NA NA NA NA NA
AJCC7 stage for TCC, n (%) Stage 0 Stage I Stage II Stage III Stage IV	0 (0%) 10 (66.7%) 2 (13.3%) 3 (20.0%) 0 (0%)	23 (22.8%) 49 (48.5%) 15 (14.9%) 12 (11.9%) 2 (2.0%)	NA NA NA NA NA	5 (11.4%) 12 (27.3%) 6 (13.6%) 10 (22.7%) 11 (25.0%)	NA NA NA NA NA	NA NA NA NA NA
HBsAg positive, *n* (%)	8 (53.3%)	30 (29.7%)	NA	NA	210 (79.2%)	124 (81.0%)
Anti-HCV positive, *n* (%)	5 (33.3%)	47 (46.5%)	NA	NA	66 (24.9%)	54 (35.3%)
*Biochemistry and hemogram*						
Total bilirubin (mg/dL) ± SD	0.7 ± 0.3	1.0 ± 0.9	NA	NA	1.3 ± 1.7	NA
AST(U/L) ± SD	44.3 ± 25.5	143.2 ± 55.3	NA	NA	76.5 ± 103.3	NA
ALT(U/L) ± SD	37.2 ± 19.0	108.3 ± 51.2	NA	NA	75.6 ± 97.5	NA
Albumin (g/dL) ± SD	4.0 ± 0.6	3.7 ± 0.6	NA	NA	3.9 ± 0.6	NA
Platelet count (×10^3^/mL) ± SD	153.0 ± 42.4	163 ± 72.0	NA	NA	NA	NA
AFP (ng/mL), median (range)	5.7 (2.4–87.2)	8.1 (2.1–20844.1)	NA	NA	33.0 (1.0–443209.0)	NA

^a^ The sequential order scores were defined according to the order of TCC diagnosed, when compared to the date of HCC. Score 1, TCC diagnosed prior to HCC; Score 2, TCC diagnosed synchronously to HCC; Score 3, TCC diagnosed metachronously to HCC. BCLC, Barcelona Clinic Liver Cancer; AJCC, The American Joint Committee on Cancer; AST, aspartate transaminase; ALT, alanine transaminase; AFP, alpha-fetoprotein; DPC, HCC/TCC double primary cancer; TES, targeted exome sequencing; RT-qPCR, real-time quantitative polymerase chain reaction; IHC, immunohistochemical staining; NA, not available.

**Table 2:** Summary of potentially pathogenic variants identified in 15 patients with HCC and TCC double primary cancers.

Symbol	SNP ID	Chr	Position	Ref	Var	NM_ID	cDNA Change	Codon Change	EXON	Protein Change	CADD Score
*DCC*	rs9951523	18	49867224	T	C	NM_005215	c.67T > C	Ttc/Ctc	1/29	p.F23L	22.3
*EML4*	rs6736913	2	42510018	A	G	NM_019063	c.847A > G	Aaa/Gaa	8/23	p.K283E	22.5
*ERCC5*	rs9514066	13	103527849	G	C	NM_000123	c.3157G > C	Gga/Cga	15/15	p.G1053R	21.5
*FN1*	rs386524617	2	216235089	C	T	NM_002026	c.6415G > A	Gtc/Atc	41/46	p.V2139I	20.3
*ITGB2*	rs235330	21	46314907	T	A	NM_000211	c.1062A > T	caA/caT	10/17	p.Q354H	21.5
*PKHD1*	rs2435322	6	51875250	A	C	NM_138694	c.5608T > G	Ttg/Gtg	35/67	p.L1870V	20.3
*ITGA9*	rs267561	3	37574951	G	A	NM_002207	c.1520G > A	gGa/gAa	14/28	p.G507E	24.4
*FN1*	rs1250259	2	216300482	T	A	NM_002026	c.44A > T	cAg/cTg	1/46	p.Q15L	22
*DPYD*	rs1801265	1	98348885	G	A	NM_000110	c.85C > T	Cgt/Tgt	2/23	p.R29C	23.2
*PLCG1*	rs753381	20	39797465	T	C	NM_002660	c.2438T > C	aTc/aCc	22/33	p.I813T	22.9
*SYNE1*	rs214976	6	152772264	A	G	NM_033071	c.3125T > C	gTa/gCa	26/146	p.V1042A	20.1
*FLT3*	rs1933437	13	28624294	G	A	NM_004119	c.680C > T	aCg/aTg	6/24	p.T227M	21.4
*SYNE1*	rs2306916	6	152647681	A	T	NM_033071	c.14830T > A	Ttg/Atg	78/146	p.L4944M	21
*PBX1*	rs2275558	1	164529120	G	A	NM_001204961	c.61G > A	Ggc/Agc	1/9	p.G21S	20.9
*CYP2D6*	rs1065852	22	42526694	G	A	NM_001025161	c.100C > T	Cca/Tca	1/8	p.P34S	23.7
*FLT4*	rs448012	5	180046344	G	C	NM_002020	c.2670C > G	caC/caG	19/30	p.H890Q	22.9
*SYNE1*	rs9479297	6	152658142	T	C	NM_033071	c.12149A > G	aAg/aGg	75/146	p.K4050R	20.4
*PTCH1*	rs357564	9	98209594	G	A	NM_000264	c.3944C > T	cCc/cTc	23/24	p.P1315L	24.9
*ERBB2*	rs1058808	17	37884037	C	G	NM_004448	c.3508C > G	Ccc/Gcc	27/27	p.P1170A	23.5
*EPHA3*	rs35124509	3	89521693	T	C	NM_005233	c.2770T > C	Tgg/Cgg	16/17	p.W924R	22.3
*ERCC5*	rs17655	13	103528002	G	C	NM_000123	c.3310G > C	Gat/Cat	15/15	p.D1104H	22.9
*CSF1R*	rs10079250	5	149450132	T	C	NM_005211	c.1085A > G	cAc/cGc	8/22	p.H362R	20.4
*LRP1B*	rs12990449	2	142567910	T	C	NM_018557	c.143A > G	cAg/cGg	2/91	p.Q48R	20.4
*DST*	rs11756977	6	56420538	C	T	NM_001144770	c.6872G > A	cGt/cAt	41/84	p.R2291H	22.9
*NIN*	rs2295847	14	51202311	G	C	NM_182946	c.5800C > G	Caa/Gaa	28/30	p.Q1934E	22.5
*PARP1*	rs1136410	1	226555302	A	G	NM_001618	c.2285T > C	gTg/gCg	17/23	p.V762A	28.1
*PIK3R1*	rs386584794	5	67588148	G	A	NM_181523	c.978G > A	atG/atA	8/16	p.M326I	20.7
*SETD2*	rs76208147	3	47162886	C	T	NM_014159	c.3240G > A	atG/atA	3/21	p.M1080I	20.7
*CRTC1*	rs3746266	19	18876309	A	G	NM_015321	c.982A > G	Acc/Gcc	9/14	p.T328A	22.5
*SYNE1*	rs76160752	6	152629631	C	T	NM_033071	c.17126G > A	cGg/cAg	90/146	p.R5709Q	22.4
*CASC5*	rs11858113	15	40914177	T	C	NM_170589	c.1793T > C	aTg/aCg	11/27	p.M598T	20.8
*CDH11*	rs35195	16	65025718	G	A	NM_001797	c.764C > T	aCg/aTg	6/13	p.T255M	25.1
*CDH11*	rs1130821	16	65022234	C	T	NM_001797	c.825G > A	atG/atA	7/13	p.M275I	24.9
*DST*	rs80260070	6	56351972	G	C	NM_001144770	c.13108C > G	Ctg/Gtg	68/84	p.L4370V	23
*EPHA3*	rs17801309	3	89521664	G	A	NM_005233	c.2741G > A	cGc/cAc	16/17	p.R914H	22.3
*EPHA7*	rs2278106	6	94120219	G	A	NM_004440	c.832C > T	Ccc/Tcc	3/17	p.P278S	23.4
*ERBB2*	rs1136201	17	37879588	A	G	NM_004448	c.1963A > G	Atc/Gtc	17/27	p.I655V	22.3
*FANCA*	rs11646374	16	89857935	G	A	NM_000135	c.1235C > T	gCg/gTg	14/43	p.A412V	21.4
*HNF1A*	rs1169288	12	121416650	A	C	NM_000545	c.79A > C	Atc/Ctc	1/10	p.I27L	23.4
*IGF2R*	rs8191754	6	160448324	C	G	NM_000876	c.754C > G	Ctg/Gtg	6/48	p.L252V	23.4
*IKBKB*	rs2272736	8	42177163	G	A	NM_001556	c.1577G > A	cGg/cAg	15/22	p.R526Q	24.4
*MTRR*	rs2287780	5	7889304	C	T	NM_002454	c.1324C > T	Cgc/Tgc	9/15	p.R442C	23.1
*ROS1*	rs1998206	6	117725448	T	G	NM_002944	c.433A > C	Act/Cct	5/43	p.T145P	23.7
*TRIP11*	rs80200454	14	92460227	C	T	NM_004239	c.5086G > A	Gaa/Aaa	15/21	p.E1696K	23.5

Chr, chromosome; Ref, reference; Var, variation; CADD, combined annotation dependent depletion.

**Table 3:** Comparisons of identified potentially pathogenic alleles in double cancers with normal population in Taiwan.

Symbol	SNP ID	Genotype		Three-Group Comparison							Two-Group Comparison				
				HCC + TCC			Normal Population			*p*	HCC + TCC		Normal Population		*p*
		Ref	Var	Var Homo	Hetero	Ref Homo	Var Homo	Hetero	Ref Homo		Var Homo	Non-Var Homo	Var Homo	Non-Var Homo	
*SYNE1*	rs9479297	T	C	**5**	**8**	**2**	4	**62**	**150**	**0.002**	**5**	**10**	**4**	**212**	**0.009**
*SYNE1*	rs76160752	A	G	**1**	**0**	**14**	**0**	**15**	**201**	**<0.001**	1	14	0	216	0.077
*EPHA*	rs17801309	G	C	**1**	**3**	**11**	**0**	**28**	**188**	**<0.001**	1	14	0	216	0.077
*TRIP11*	rs80200454	C	T	**1**	**0**	**14**	**0**	**19**	**197**	**<0.001**	1	14	0	216	0.077
*ITGA9*	rs267561	T	A	14	1	0	151	54	11	0.148	14	1	151	65	0.100
*SETD2*	rs76208147	A	C	2	3	10	5	52	159	0.055	2	13	5	211	0.103
*PBX1*	rs2275558	G	A	8	5	2	82	101	33	0.488	8	7	82	134	0.238
*CRTC1*	rs3746266	T	A	2	6	7	8	64	144	0.113	2	13	8	208	0.264
*SYNE1*	rs214976	G	A	11	4	0	127	75	14	0.42	11	4	127	89	0.267
*EPHA3*	rs35124509	T	C	2	6	7	8	72	136	0.15	2	13	8	208	0.264
*CYP2D6*	rs1065852	A	G	7	3	5	71	99	46	0.147	7	8	71	145	0.275
*FLT3*	rs1933437	G	A	10	4	1	116	84	16	0.609	10	5	116	100	0.330
*FLT4*	rs448012	A	T	6	2	7	59	93	64	0.077	6	9	59	157	0.447
*ERCC5*	rs17655	G	A	2	6	7	55	94	67	0.377	2	13	55	161	0.457
*SYNE1*	rs2306916	G	A	9	5	1	148	63	5	0.534	9	6	148	68	0.467
*NIN*	rs2295847	G	C	2	7	6	53	117	46	0.216	2	13	53	163	0.502
*CDH11*	rs1130821	T	C	1	8	6	35	108	73	0.604	1	14	35	181	0.537
*MTRR*	rs2287780	G	A	1	4	10	3	65	148	0.314	1	14	3	213	0.623
*FN1*	rs1250259	C	G	14	1	0	184	30	2	0.673	14	1	184	32	0.624
*DPYD*	rs1801265	T	C	13	2	0	184	31	1	0.959	13	2	184	32	0.624
*LRP1B*	rs12990449	G	C	2	5	8	48	93	75	0.337	2	13	48	168	0.628
*CASC5*	rs11858113	T	C	1	5	9	12	61	143	0.887	1	14	12	204	0.690
*ERBB2*	rs1136201	T	C	1	3	11	4	48	164	0.462	1	14	4	212	0.748
*IKBKB*	rs2272736	C	T	1	5	9	4	40	172	0.152	1	14	4	212	0.748
*IGF2R*	rs8191754	G	C	1	4	10	10	70	136	0.862	1	14	10	206	0.788
*FANCA*	rs11646374	A	G	1	6	8	10	75	131	0.836	1	14	10	206	0.788
*PLCG1*	rs753381	G	A	11	4	0	144	65	7	0.728	11	4	144	72	0.805
*CSF1R*	rs10079250	C	T	2	5	8	42	107	67	0.203	2	13	42	174	0.808
*PARP1*	rs1136410	A	G	2	8	5	42	98	76	0.786	2	13	42	174	0.808
*CDH11*	rs35195	C	T	1	3	11	5	76	135	0.328	1	14	5	211	0.853
*ERBB2*	rs1058808	T	C	3	5	7	32	104	80	0.536	3	12	32	184	0.866
*HNF1A*	rs1169288	G	A	1	7	7	25	109	82	0.735	1	14	25	191	0.874
*DST*	rs11756977	C	T	2	7	6	34	84	98	0.836	2	13	34	182	0.905
*DST*	rs80260070	G	C	1	4	10	6	62	148	0.695	1	14	6	210	0.944
*EPHA7*	rs2278106	G	A	1	4	10	7	58	151	0.78	1	14	7	209	0.977
*ROS1*	rs1998206	G	A	1	3	11	7	65	144	0.59	1	14	7	209	0.977
*PTCH1*	rs357564	A	G	4	8	3	66	104	46	0.924	4	11	66	150	0.979
*DCC*	rs9951523	G	A	15	0	0	216	0	0	NA	15	0	216	0	NA
*EML4*	rs6736913	A	C	15	0	0	216	0	0	NA	15	0	216	0	NA
*ERCC5*	rs9514066	C	G	15	0	0	216	0	0	NA	15	0	216	0	NA
*FN1*	rs386524617	G	A	15	0	0	216	0	0	NA	15	0	216	0	NA
*ITGB2*	rs235330	C	T	15	0	0	216	0	0	NA	15	0	216	0	NA
*PKHD1*	rs2435322	T	G	15	0	0	216	0	0	NA	15	0	216	0	NA
*PIK3R1*	rs386584794	C	T	2	2	11	NA	NA	NA	NA	2	13	NA	NA	NA

The *p* values were derived by using two-tailed chi-square analysis with Yate’s correction. Ref, reference; Var, variation; Var homo, variation homozygote; Hetero, heterozygote; Ref homo, reference homozygote.

**Table 4:** The *SYNE1*-rs9479297 genotypes were associated with HCC and TCC occurrence in Taiwan.

Population in Taiwan	*SYNE1*-rs9479297 Genotypes			*p*	*SYNE1*-rs9479297 Genotypes		*p*
	CC	CT	TT		TT	Non-TT	
HCC + TCC patients (*n* = 101)	72	17	12		12	89	
TCC patients (*n* = 44)	34	10	0	0.051	0	44	0.039
HCC patients (*n* = 265)	187	67	11	0.010	11	254	0.006
Non-TCC and non-HCC patients (*n* = 153)	113	38	2	0.001	2	146	<0.001
Normal population (*n* = 216)	150	62	4	<0.001	4	212	<0.001

The *p* values were derived by using two-tailed chi-square analysis with Yate’s correction.

**Table 5:** Comparison of baseline characteristics between HCC/TCC DPC patients genotyped as rs9479297-TT and non-TT.

Characteristics	rs9479297-TT	rs9479297-Non-TT	*p*
Gender, male, *n* (%)	8 (58.3%)	57 (70.4%)	0.5005
HCC diagnosed Ages (years) ± SD	61.9 ± 8.5	67.5 ± 8.1	**0.0358**
TCC diagnosed Ages (years) ± SD	60.3 ± 7.1	67.2 ± 8.1	**0.0067**
Sequential order score ± SD ^a^	1.8 ± 0.8	1.9 ± 0.8	0.5352
Body weight ± SD	61.1 ± 11.4	63.3 ± 11.3	0.7355
Body height ± SD	161.1 ± 7.7	161.2 ± 7.1	0.9756
Heavy smoker, *n* (%)	4 (33.3%)	37 (41.6%)	0.8161
BCLC stage for HCC, *n* (%) Stage 0 Stage A Stage B Stage C Stage D	0 (0.0%) 5 (41.7%) 3 (25.0%) 4 (33.3%) 0 (0.0%)	%15 (16.9%) 18 (20.2%) 17 (19.1%) 38 (42.7%) 1 (1.1%)	0.2675 0.1950 0.9239 0.7598 0.7121
AJCC7 stage for TCC, *n* (%) Stage 0 Stage I Stage II Stage III Stage IV	3 (25.0%) 6 (50.0%) 2 (16.7%) 1 (8.3%) 0 (0.0%)	20 (22.5%) 43 (48.3%) 13 (14.6%) 11 (12.4%) 2 (2.2%)	0.8446 0.9127 0.8506 0.6857 0.5999
HBsAg positive, *n* (%)	2 (16.7%)	28 (31.4%)	0.4738
Anti-HCV positive, *n* (%)	7 (58.3%)	40 (44.9%)	0.5723
Biochemistry and hemogram			
Total bilirubin (mg/dL) ± SD	1.3 ± 1.8	0.9 ± 1.3	0.5570
AST(U/L) ± SD	142.3 ± 44.5	144.5 ± 55.7	0.9591
ALT(U/L) ± SD	141.4 ± 63.6	89.5 ± 41.6	0.3968
Albumin (g/dL) ± SD	3.6 ± 0.4	3.8 ± 0.7	0.2487
Platelet count (×10^3^/mL) ± SD	179.2 ± 84.3	157.2 ± 70.0	0.2459
AFP (ng/mL), median (range)	7.9 (2.7–488.1)	10.7 (2.1–20844.1)	0.6931

^a^ The sequential order scores were defined according to the order of TCC diagnosed, when compared to the date of HCC. Score 1, TCC diagnosed prior to HCC; Score 2, TCC diagnosed synchronously to HCC; Score 3, TCC diagnosed metachronously to HCC. BCLC, Barcelona Clinic Liver Cancer; AJCC, The American Joint Committee on Cancer; AST, aspartate transaminase; ALT, alanine transaminase; AFP, alpha-fetoprotein.

## Data Availability

The data presented in this study are available on request from the corresponding author.

## Supplementary Materials

The following are available online at https://www.mdpi.com/article/10.3390/biomedicines9121819/s1, Figure S1: The schematic image illustrated the cohorts enrolled in this study for the indicated analyses, Figure S2: Frequency and distribution of candidate germline variants contributing to HCC/TCC co-occurrence, Figure S3: Clinical outcomes in patients with TCC or DPC in relationship to *SYNE1*-rs9479297 genotypes, Table S1: Genes included in targeted exome sequencing in this study.
