# Supplementary material for: SYNE1 Exonic Variant rs9479297 Contributes to Concurrent Hepatocellular and Transitional Cell Carcinoma Double Primary Cancer
Source: Biomedicines. 2021 Dec 2;9(12):1819. doi: 10.3390/biomedicines9121819 (PMC8698502; doi:10.3390/biomedicines9121819)
Supplement: Supplementary file 1 [file biomedicines-09-01819-s001.zip › biomedicines-1493703-supplementary.pdf]

biomedicines

Article

SYNE1 Exonic Variant rs9479297 Contributes to Concurrent Hepatocellular and Transitional Cell Carcinoma Double Primary Cancer

Yu-De Chu [1], Kwong-Ming Kee [2], Wey-Ran Lin [1,3], Ming-Wei Lai [1,4], Sheng-Nan Lu [2], Wen-Hung Chung [5], See-Tong Pang [6] and Chau-Ting Yeh [1,7,*]

1 Liver Research Center, Chang Gung Memorial Hospital, Taoyuan 333, Taiwan; yudechu19871003@gmail.com (Y.-D.C.); victor.wr.lin@gmail.com (W.-R.L.); mingweilai@gmail.com (M.-W.L.)
2 Division of Hepatogastroenterology, Department of Internal Medicine, Kaohsiung Chang Gung Memorial Hospital, Kaohsiung 833, Taiwan; kee.kkm@gmail.com (K.-M.K.); juten@ms17.hinet.net (S.-N.L.)
3 Department of Hepatology and Gastroenterology, Linkou Chang Gung Memorial Hospital, Taoyuan 333, Taiwan
4 Division of Pediatric Gastroenterology Department of Pediatrics, Linkou Chang Gung Memorial Hospital, Taoyuan 333, Taiwan
5 Whole-Genome Research Core Laboratory of Human Diseases, Chang Gung Memorial Hospital, Keelung 204, Taiwan; wenhungchung@yahoo.com
6 Division of Urology, Department of Surgery, Linkou Chang Gung Memorial Hospital, Taoyuan 333, Taiwan; jacobpang@cgmh.org.tw
7 Molecular Medicine Research Center, Chang Gung University, Taoyuan 333, Taiwan
* Correspondence: chautingy@gmail.com

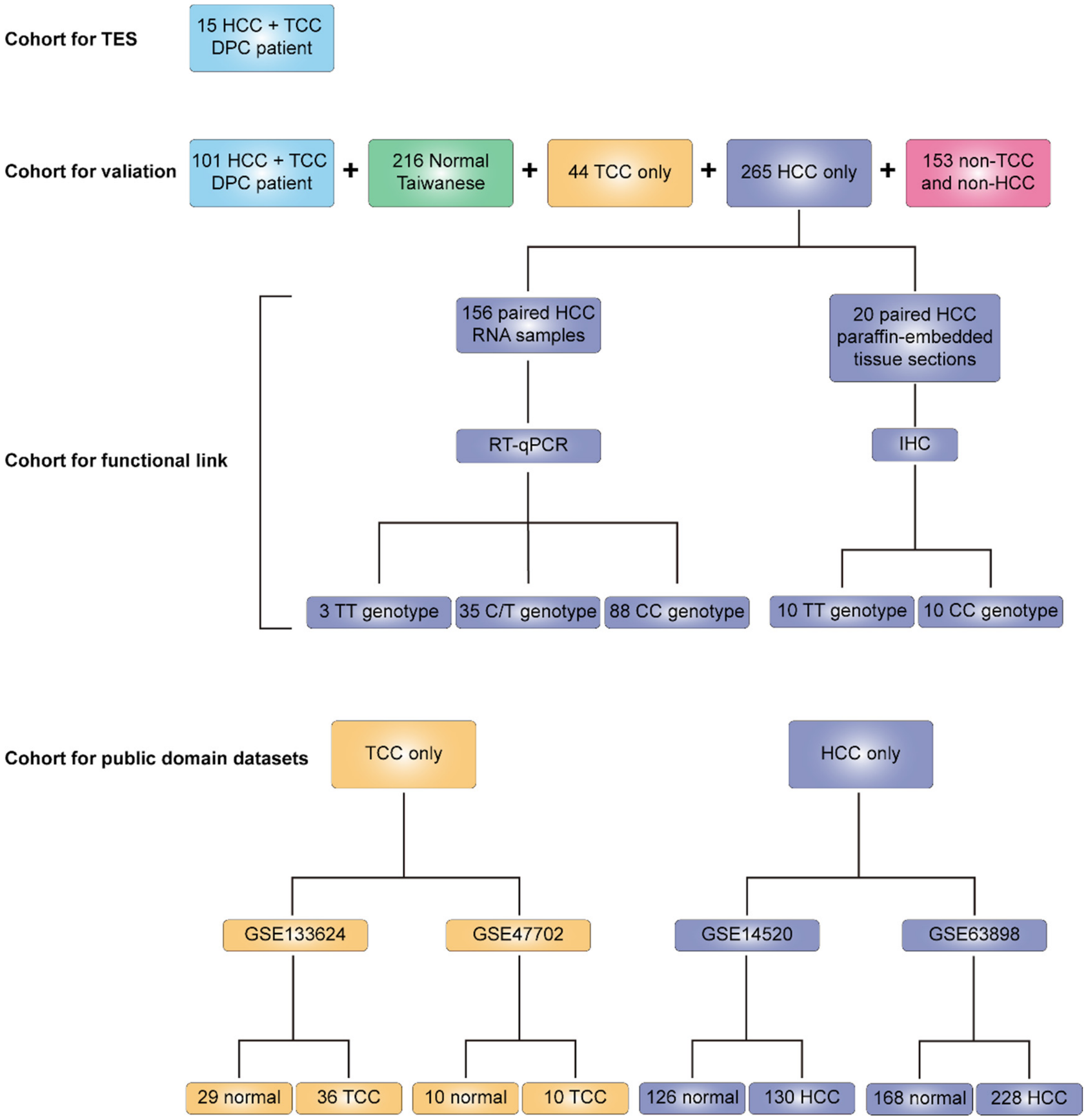

Figure S1. The schematic image illustrated the cohorts enrolled in this study for the indicated analyses.

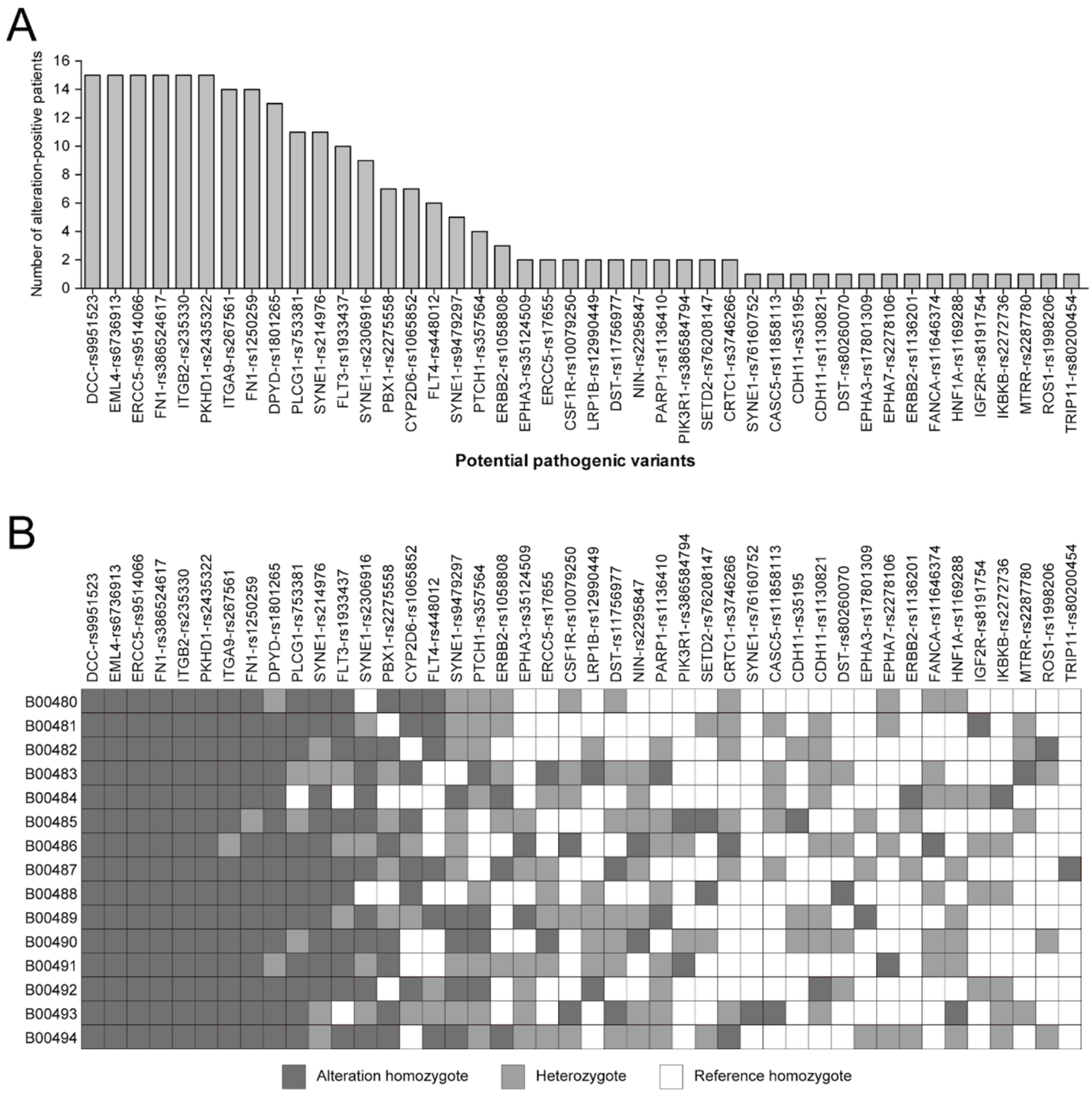

Figure S2. Frequency and distribution of candidate germline variants contributed to HCC/TCC co-occurrence. (A) The alteration-positive counts of each variation site among 15 HCC/TCC DPC patients submitted to TES. (B) The details of each variant identified in individual patient.

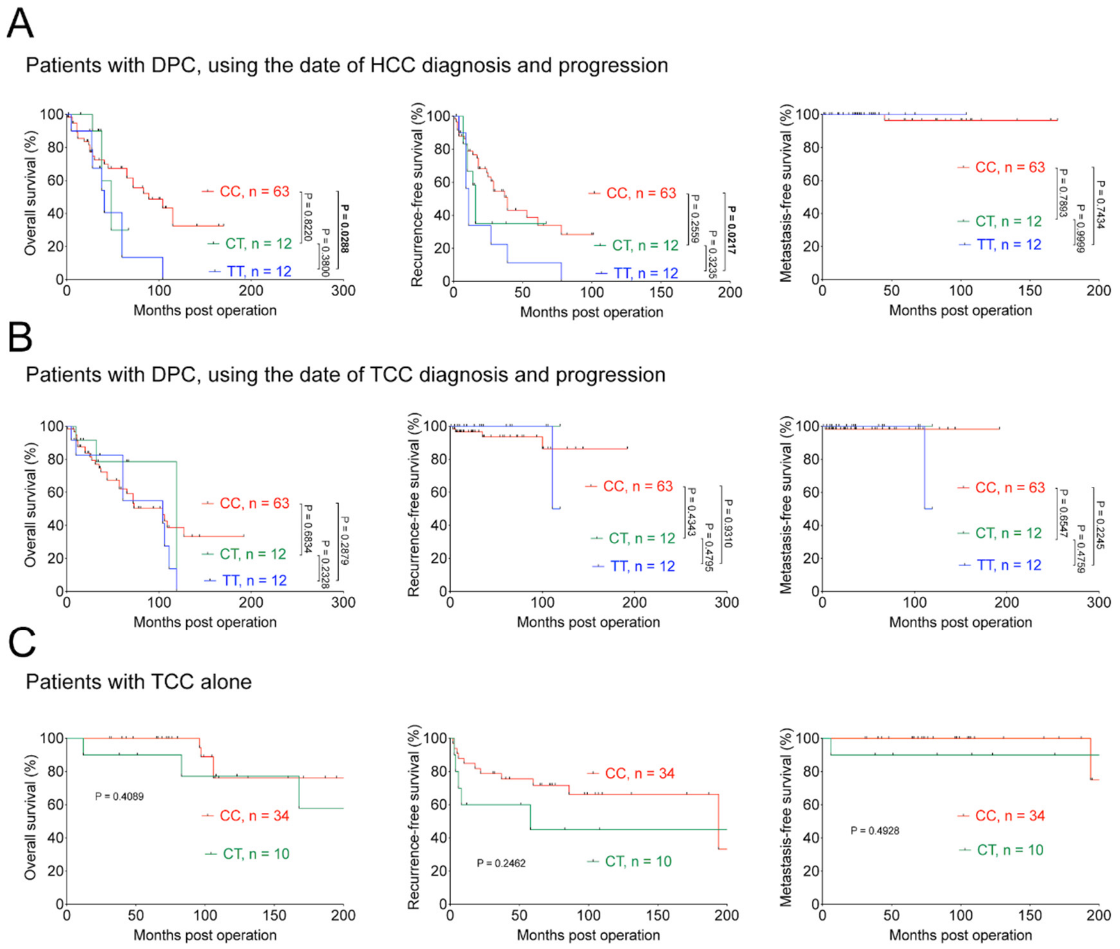

Figure S3. Clinical outcomes in patients with TCC or DPC in relationship to *SYNE1*-rs9479297 genotypes. The Kaplan-Meyer plots illustrated the OS (left), RFS (middle), and MFS (right) in 87 DPC patients, either (A) using the of HCC diagnosed and progressed or (B) using the of TCC diagnosed and progressed. (C) The Kaplan-Meyer plots illustrated the OS (left), RFS (middle), and MFS (right), in 44 patients with TCC. Red lines, *SYNE1*-rs9479297-CC genotype; Green lines, *SYNE1*-rs9479297-CT genotype. Blue lines, *SYNE1*-rs9479297-TT genotype, None of the TCC patients had *SYNE1*-rs9479297-TT genotype.

Table S1. Genes included in targeted exome sequencing in this study.

ABL1	BRIP1	DDIT3	FLT1	ITGA9	MET	PALB2	RALGDS	TCF12
ABL2	BTK	DDR2	FLT3	ITGB2	MITF	PARP1	RARA	TCF3
ACVR2A	BUB1B	DEK	FLT4	ITGB3	MLH1	PAX3	RB1	TCF7L1
ADAMTS20	CARD11	DICER1	FN1	JAK1	MLLT10	PAX5	RECOL4	TCFL2
AFF1	CASC5	DNMT3A	FOXL2	JAK2	MMP2	PAX7	REL	TCL1A
AFF3	CBL	DPYD	FOXO1	JAK3	MN1	PAX8	RET	TET1
AKAP9	CCND1	DST	FOXO3	JUN	MPL	PBRM1	RHOH	TET2
AKT1	CCND2	EGFR	FOXOP1	KAT6A	MRE11A	PBX1	RNASEL	TFE3
AKT2	CCNE1	EML4	FOXOP4	KAT6B	MSH2	PDE4DIP	RNF2	TGFBR2
AKT3	CD79A	EP300	FZR1	KDM5C	MSH6	PDGFB	RNF213	TGM7
ALK	CD79B	EP400	G6PD	KDM6A	MTOR	PDGFRA	ROS1	THBS1
AMER1	CDC73	EPHA3	GATA1	KDR	MTR	PDGFRB	RPS6KA2	TIMP3
APC	CDH1	EPHA7	GATA2	KEAP1	MTRR	PER1	RRM1	TLR4
AR	CDH11	EPHB1	GATA3	KIT	MUC1	PGAP3	RUNX1	TLX1
ARID1A	CDH2	EPHB4	GDNF	KLF6	MUTYH	PHOX2B	RUNX1T1	TNFAIP3
ARID2	CDH20	EPHB6	GNA11	KMT2B	MYB	PIK3C2B	SAMD9	TNFRSF14
ARNT	CDH5	ERBB2	GNAQ	KMT2C	MYC	PIK3CA	SBDS	TNK2
ASXL1	CDK12	ERBB3	GNAS	KMT2D	MYCL	PIK3CB	SDHA	TOP1
ATF1	CDK4	ERBB4	GPR124	KRAS	MYCN	PIK3CD	SDHB	TP53
ATM	CDK6	ERCC1	GRM8	LAMP1	MYD88	PIK3CG	SDHC	TPR
ATR	CDK8	ERCC2	GUCY1A2	LCK	MYH11	PIK3R1	SDHD	TRIM24
ATRX	CDKN2A	ERCC3	HCAR1	LIFR	MYH9	PIK3R2	SEPT9	TRIM33
AURKA	CDKN2B	ERCC4	HIF1A	LPHN3	NBN	PIM1	SETD2	TRIP11
AURKB	CDKN2C	ERCC5	HLF	LPP	NCOA1	PKHD1	SF3B1	TRRAP
AURKC	CEBPA	ERG	HNF1A	LRP1B	NCOA2	PLAG1	SGK1	TSC1
AXL	CHEK1	ESR1	HOOK3	LTF	NCOA4	PLOG1	SH2D1A	TSC2
BAI3	CHEK2	ETS1	HRAS	LTK	NF1	PLEKHG5	SMAD2	TSHR
BAP1	CIC	ETV1	HSP90AA1	MAF	NF2	PML	SMAD4	UBR5
BCL10	CKS1B	ETV4	HSP90AB1	MAFB	NFE2L2	PMS1	SMARCA4	UGT1A1
BCL11A	CMPK1	EXT1	ICK	MAGEA1	NFKB1	PMS2	SMARCB1	USP9X
BCL11B	COL1A1	EXT2	IDH1	MAGI1	NFKB2	POT1	SMO	VHL
BCL2	CRBN	EZH2	IDH2	MALT1	NIN	POU5F1	SMUG1	WAS
BCL2L1	CREB1	FANCA	IGF1R	MAML2	NKX2-1	PPARG	SOCS1	WHSC1
BCL2L2	CREBBP	FANCC	IGF2	MAP2K1	NLRP1	PPP2R1A	SOX11	WRN
BCL3	CRKL	FANCD2	IGF2R	MAP2K2	NOTCH1	PRDM1	SOX2	WT1
BCL6	CRTC1	FANCF	IKBKB	MAP2K4	NOTCH2	PRKAR1A	SRC	XPA
BCL9	CSF1R	FANCG	IKBKE	MAP3K7	NOTCH4	PRKDC	SSX1	XPC
BCR	CSMD3	FAS	IKZF1	MAPK1	NPM1	PSIP1	STK11	XOP1
BIRC2	CTNNA1	FBXW7	IL2	MAPK8	NRAS	PTCH1	STK36	XRCC2
BIRC3	CTNNB1	FGFR1	IL21R	MARK1	NSD1	PTEN	SUFU	ZNF384
BIRC5	CYLD	FGFR2	IL6ST	MARK4	NTRK1	PTGS2	SYK	ZNF521
BLM	CYP2C19	FGFR3	IL7R	MBD1	NTRK3	PTPN11	SYNE1	
BLNK	CYP2D6	FGFR4	ING4	MCL1	NUMA1	PTPRD	TAF1	
BMPR1A	DAXX	FH	IRF4	MDM2	NUP214	PTPRT	TAF1L	
BRAF	DCC	FLCN	IRS2	MDM4	NUP98	RAD50	TAL1	
BRD3	DDB2	FLI1	ITGA10	MEN1	PAK3	RAF1	TBX22	
